# Medical insurance coverage and its associated factors among children in urban and rural Chongqing, China

**DOI:** 10.1093/inthealth/ihaf057

**Published:** 2025-05-27

**Authors:** Caihui Hu, Jingyu Chen, Lanling Chen, Xinyuan Yao, Shunqing Luo, Xiaoping Jiang, Lan Chen, Fengming Wang, Jie Li, Jian Liu, Shihai Zheng, Xiaohua Liang

**Affiliations:** Department of Clinical Epidemiology and Biostatistics, Children's Hospital of Chongqing Medical University, National Clinical Research Center for Child Health and Disorders, Ministry of Education Key Laboratory of Child Development and Disorders, Chongqing Key Laboratory of Pediatric Metabolism and Inflammatory Diseases, Chongqing 400014, China; Department of Ultrasound Medicine, Children's Hospital of Chongqing Medical University, Chongqing, China; Department of Clinical Epidemiology and Biostatistics, Children's Hospital of Chongqing Medical University, National Clinical Research Center for Child Health and Disorders, Ministry of Education Key Laboratory of Child Development and Disorders, Chongqing Key Laboratory of Pediatric Metabolism and Inflammatory Diseases, Chongqing 400014, China; Hematology and Oncology, Children's Hospital of Chongqing Medical University, Chongqing, China; Department of General Medicine, Children's Hospital of Chongqing Medical University, Chongqing, China; Department of Nursing, Children's Hospital of Chongqing Medical University, Chongqing, China; Department of Clinical Epidemiology and Biostatistics, Children's Hospital of Chongqing Medical University, National Clinical Research Center for Child Health and Disorders, Ministry of Education Key Laboratory of Child Development and Disorders, Chongqing Key Laboratory of Pediatric Metabolism and Inflammatory Diseases, Chongqing 400014, China; Department of Clinical Epidemiology and Biostatistics, Children's Hospital of Chongqing Medical University, National Clinical Research Center for Child Health and Disorders, Ministry of Education Key Laboratory of Child Development and Disorders, Chongqing Key Laboratory of Pediatric Metabolism and Inflammatory Diseases, Chongqing 400014, China; Department of Clinical Epidemiology and Biostatistics, Children's Hospital of Chongqing Medical University, National Clinical Research Center for Child Health and Disorders, Ministry of Education Key Laboratory of Child Development and Disorders, Chongqing Key Laboratory of Pediatric Metabolism and Inflammatory Diseases, Chongqing 400014, China; Department of Clinical Epidemiology and Biostatistics, Children's Hospital of Chongqing Medical University, National Clinical Research Center for Child Health and Disorders, Ministry of Education Key Laboratory of Child Development and Disorders, Chongqing Key Laboratory of Pediatric Metabolism and Inflammatory Diseases, Chongqing 400014, China; Department of Medical Laboratory, Chongqing Liangjiang New Area People's Hospital, Chongqing 401121, China; Department of Clinical Epidemiology and Biostatistics, Children's Hospital of Chongqing Medical University, National Clinical Research Center for Child Health and Disorders, Ministry of Education Key Laboratory of Child Development and Disorders, Chongqing Key Laboratory of Pediatric Metabolism and Inflammatory Diseases, Chongqing 400014, China

**Keywords:** associated factors, basic medical insurance, children, commercial medical insurance, medical insurance

## Abstract

**Background:**

Children face a heavy disease burden, while healthcare utilization remains low. This study seeks to assess the proportions of children in Chongqing covered by medical insurance and identify associated factors.

**Methods:**

From March to June 2019, a stratified cluster sampling was employed to cover 4705 participants in Chongqing's urban and rural districts. In a cross-sectional survey, univariate and multivariate mixed logistic regression analysis were performed to explore the determinants of medical insurance enrolment.

**Results:**

The participation rates of basic medical insurance (BMI) were 83.29%, 85.29% and 81.11% in total, urban areas and rural areas, respectively. For commercial medical insurance (CMI), the corresponding rates were 29.78%, 34.95% and 24.11%, respectively. After adjusting for covariates, younger child age, better quality of life and higher annual household income were associated with a higher BMI participation rate. Conversely, children with asthma had lower odds of BMI coverage. For CMI, childhood obesity was a risk factor for being uninsured, while higher parental education, rhinitis, annual family income >150 000 RMB, caesarean section history and maternal gestational diabetes significantly increased the likelihood of CMI enrolment.

**Conclusions:**

In summary, universal health insurance coverage for children in Chongqing remains unfulfilled. To ameliorate the gaps and inequalities in children's insurance, sustained efforts are necessary, including improving household economic conditions, enhancing parental education levels and focusing on children's physical health. Therefore, policy supports should be enhanced, especially for economically disadvantaged rural areas in southwestern China.

## Introduction

In 2016, global years lost due to disability for children ages 0–19 y were approximately 130 million, with anaemia, congenital abnormalities and asthma as major contributors.^1^ In China, the standardized disability-adjusted life years (DALYs) for children <5 y of age were 16 479.01 per 100 000 in 2019, with neonatal preterm birth and congenital heart abnormalities as the leading causes.^2^ These statistics underscore the persistent global and national concerns regarding child health. In 2021, China launched the Healthy Children Program under the Child Development Program to prioritize the health of key populations, including women and children, at the national level.^3^

Health insurance enrolment improves access to care and health outcomes by reducing out-of-pocket costs. Studies have shown that childhood insurance is associated with lower mortality, fewer chronic diseases,^4^ higher birthweight,^5^ improved education, greater economic gains,^6^ cognitive development and reduced reliance on government aid in later life.^7^ However, cancer patients without insurance were more likely to present with stage 3 and 4 tumours at the time of initial diagnosis, face high-risk pathology grades and experience poorer survival rates.^8^ Therefore, it is crucial to investigate the status of children's insurance coverage and improve the low participation rate. In 2015, the World Health Organization reported that 400 million people lacked access to basic health services and 6% of the population in low- and middle-income countries were pushed into extreme poverty due to health costs.^9^ In 2016, the participation rate of Chinese children in basic medical insurance (BMI) was 88.8%,^10^ with rural coverage at 55.03%.^11^ By September 2022, the national BMI participation rate reached 95%.^12^ In contrast, the USA implemented the Child Health Insurance Program and Affordable Care Act, reducing the uninsured rate among children from 8.3% in 2016 to 3.7% in 2022, despite disruptions from the coronavirus disease 2019 (COVID-19) pandemic.^13^ These disparities underscore the need for continued efforts to improve child health insurance coverage in China.

A large US study found that children from mixed-status or non-citizen families had significantly higher uninsured rates than those from citizen families, with the disparity widening over time.^14^ Research also highlighted that parental education level, employment, rural residence,^15^ low income, older age and physical conditions^16^ partially accounted for children being uninsured, while the number of siblings and parental marital status had no bearing on insurance coverage.^17^ In China, infants and urban children with less-educated mothers or a migrant parent were less likely to be insured.^18^ Additionally, Other studies showed that children's insurance status was closely tied to parental insurance coverage.^19,20^ Overall, socio-economic factors, particularly income and education, were independent contributors to insurance coverage, likely influencing affordability, awareness of insurance benefits and access to healthcare.^21–23^ Moreover, family structures and the presence of chronic illnesses or disabilities can also influence insurance coverage in children.^24^

Research from Hisle-Gorman et al.^25^ reported that poor parental health adversely affected family dynamics and children's healthcare access. Perinatal factors such as prematurity, caesarean delivery and maternal gestational conditions can influence children's long-term health outcomes, including neurodevelopmental impairments and elevated risk of various systemic diseases.^26–29^ An Indian study indicated that adverse perinatal factors significantly reduced children's health-related quality of life.^30^ Based on these findings, we hypothesize that both parental medical history and perinatal factors are closely related to children's participation in medical insurance. This cross-sectional study examines the influence of socio-economic, health, education and perinatal factors on children's participation in BMI and CMI in southwestern China, a region with underdeveloped economic conditions. It also explores participation differences between rural and urban regions in Chongqing, contributing to supplementing existing research on children's medical insurance in southwestern China and improving enrolment rates.

## Methods

### Study participants

**Figure 1.**
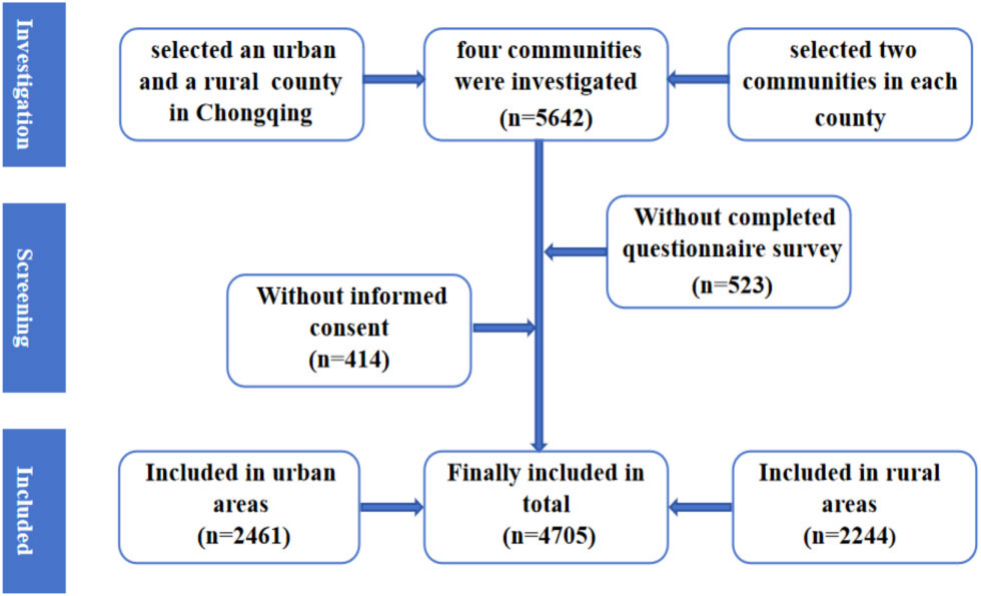
Research object inclusion process.

This study employed a two-stage stratified cluster sampling method in Chongqing, China, to select participants from March to June 2019. First, an urban county and a rural county in Chongqing were randomly chosen. Then, two communities were randomly selected from each county and a total of four communities were finally included. Finally, all the children residing in the recruited communities and attending grades 5 and 6 in primary school were included in our study. This cross-sectional study was designed to explore the status and related factors of children's enrolment in medical insurance. The inclusion criteria were residing in the survey area for at least 6 months, having a guardian capable of completing the questionnaire, age 9–14 y (in grade 5 or 6) and both the children and their guardians signed a written informed consent form. This study was approved by the Institutional Review Board of Children's Hospital of Chongqing Medical University (no. 2013-86). The sample screening flow chart is shown in Figure 1.

### Variables

A questionnaire survey was conducted in the selected communities to collect data, with all questionnaires completed under the supervision of trained researchers. This study focused on children's enrolment in medical insurance programs (BMI and CMI) and explored its associations with various factors, including demographic variables, socio-economic status (such as annual household income, quality of life, the number of children in a family, parental education level and parental physical condition), the physical health conditions in children (such as cold in the past month, asthma, rhinitis, congenital heart disease and urolithiasis), parental medical history (including hypertension, diabetes, hepatitis B and urolithiasis) and perinatal factors (including mode of delivery, full-term or preterm birth, gestational hypertension and gestational diabetes).

### Statistics analysis

Incomplete questionnaires were excluded. Completed questionnaires were entered into an Access database (Microsoft, Redmond, WA, USA). Statistical analysis was performed using R version 4.3.3 (R Foundation for Statistical Computing, Vienna, Austria). Normally distributed variables were expressed as mean±standard deviation (SD) and differences between rural and urban groups were analysed using the Student's t-test. Categorical variables, presented as percentages, were assessed using the χ^2^ test or Fisher's exact test. In addition, the univariate mixed logistic regression analysis, with urban/rural areas as a level variable, was performed to estimate the correlation between medical insurance enrolment and various variables, including socio-economic status, demographic characteristics, physical condition, family situation and perinatal factors in children. Subsequently, all significant variables were incorporated into a multivariate mixed logistic regression model to evaluate their effects on the participation rates of BMI and CMI.

## Results

### General characteristics

The general characteristics of the study population are presented in Table 1. These characteristics include participant’s age, gender, health insurance participation status, annual household income, living standards, parental education level, children's health status, parental medical history, delivery method and maternal pregnancy-related medical history. A total of 4705 children were included in this study (2428 males and 2277 females). The mean age of the participants was 11.75±0.65 y and 47.69% (2244/4705) lived in rural areas. The participation rates of BMI in total, urban areas and rural areas were 83.29%, 85.29% and 81.13%, respectively. Similarly, the participation rates of CMI in total, urban areas and rural areas were 29.78%, 34.95% and 24.11%, respectively. Compared with children residing in urban areas, those from rural areas had lower participation rates in both BMI and CMI, as well as lower parental education levels and annual household incomes (all p<0.001). Additionally, rural children exhibited a lower prevalence of conditions such as colds, asthma and rhinitis compared with urban children. The rates of children combined with preterm birth, caesarean delivery and maternal gestational diabetes were also lower among rural children (all p<0.05). However, there was no statistical difference in the parental disease history between rural and urban children.

**Table 1.** Characteristics of the study population in urban and rural areas.

Variables	Total	Urban	Rural	p-Value
Population, n (%)	4705	2461 (52.31)	2244 (47.69)	
Age (years), mean (SD)	11.75 (0.65)	11.61 (0.60)	11.90 (0.68)	**<0.001**
Gender, n (%)				0.501
Male	2428 (51.60)	1282 (52.09)	1146 (51.07)	
Female	2277 (48.40)	1179 (47.91)	1098 (48.93)	
Basic medical insurance, n (%)				0.948
No	786 (16.71)	362 (14.71)	424 (18.89)	
Yes	3919 (83.29)	2099 (85.29)	1820 (81.11)	
Commercial medical insurance, n (%)				**<0.001**
No	3304 (70.22)	1601 (65.05)	1703 (75.89)	
Yes	1401 (29.78)	860 (34.95)	541 (24.11)	
Number of children, n (%)				**<0.001**
1	1642 (44.43)	1239 (59.03)	403 (25.23)	
2	1884 (50.97)	798 (38.02)	1086 (68.00)	
≥3	170 (4.60)	62 (2.95)	108 (6.76)	
Mother's education (years), n (%)				**<0.001**
≤9	1359 (37.67)	548 (26.81)	811 (51.85)	
9–12	1214 (33.65)	734 (35.90)	480 (30.69)	
>12	1035 (28.67)	762 (37.29)	273 (17.45)	
Father's education (years), n (%)				**<0.001**
≤9	1077 (30.40)	437 (21.43)	640 (42.52)	
9–12	1269 (35.80)	730 (35.80)	539 (35.81)	
>12	1198 (33.80)	872 (42.77)	326 (21.67)	
Quality of life, n (%)				0.368
Worse	208 (5.08)	115 (4.93)	93 (5.28)	
Poor	457 (11.16)	250 (10.72)	207 (11.75)	
Medium	2246 (54.86)	1293 (55.42)	953 (54.12)	
Good	872 (21.30)	510 (21.86)	362 (20.56)	
Better	311 (7.60)	165 (7.07)	146 (8.29)	
Annual household income (RMB/y), n (%)				**<0.001**
<25 000	609 (18.29)	275 (14.84)	334 (22.63)	
25 001–50 000	714 (21.45)	327 (17.65)	387 (26.22)	
50 001–100 000	858 (25.77)	485 (26.17)	373 (25.27)	
100 001–150 000	510 (15.32)	447 (24.12)	191 (12.94)	
>150 000	638 (19.16)	191 (10.31)	97 (6.57)	
Weight, n (%)				0.136
Normal or overweight	3813 (92.21)	2151 (91.65)	1662 (92.95)	
Obese	322 (7.79)	196 (8.35)	126 (7.05)	
Children's history, n (%)				
Have a cold in the last month	1379 (32.55)	779 (34.02)	600 (30.82)	**0.029**
Asthma	177 (4.48)	114 (5.53)	63 (3.33)	**0.001**
Congenital heart disease	16 (0.40)	8 (0.39)	8 (0.42)	0.864
Rhinitis	745 (18.85)	505 (24.50)	240 (12.68)	**<0.001**
Urolithiasis	9 (0.23)	8 (0.39)	1 (0.05)	0.061
Maternal history, n (%)				
Hypertension	57 (1.58)	31 (1.56)	26 (1.60)	0.914
Diabetes	23 (0.64)	15 (0.75)	8 (0.49)	0.327
Hepatitis B	55 (1.52)	30 (1.51)	25 (1.54)	0.935
Urolithiasis	59 (1.63)	32 (1.61)	27 (1.66)	0.894
Other	88 (2.43)	49 (2.46)	39 (2.40)	0.914
Father's history, n (%)				
Hypertension	172 (4.84)	102 (5.23)	70 (4.36)	0.240
Diabetes	59 (1.66)	34 (1.74)	25 (1.56)	0.694
Hepatitis B	88 (2.47)	56 (2.87)	32 (2.00)	0.104
Urolithiasis	139 (3.91)	85 (4.35)	54 (3.37)	0.140
Other	102 (2.87)	54 (2.77)	48 (2.99)	0.762
Preterm birth, n (%)	307 (8.04)	221 (10.64)	86 (4.94)	**<0.001**
Caesarean section, n (%)	2388 (60.05)	1408 (65.25)	980 (53.88)	**<0.001**
Gestational hypertension, n (%)	82 (2.31)	51 (2.49)	31 (2.06)	0.430
Gestational diabetes, n (%)	41(1.16)	34 (1.66)	7 (0.47)	**0.001**

Significant values in bold.

### Univariate analysis to assess the associated factors of health insurance

Table 2 presents the association between various factors and enrolment in BMI, analysed using a univariate mixed logistic model. The likelihood of children participating in BMI decreased with age (odds ratio [OR] 0.776 [95% confidence interval {CI} 0.647 to 0.930]). Conversely, children whose fathers had >12 y of education were more likely to be enrolled in BMI (OR 0.1.502 [95% CI 1.052 to 2.144]). Additionally, children from families with an annual household income >150 000 renminbi (RMB) were 2.317 times more likely to participate in BMI (OR 2.317 [95% CI 1.430 to 3.754]).

**Table 2.** Univariate mixed logistic regression analysis of BMI.

Variables	β	OR (95% CI)	p-Value
Age	−0.253	0.776 (0.647 to 0.930)	**0.006**
Female	−0.077	0.926 (0.717 to 1.195)	0.554
Number of children			
1		1	
2	0.071	1.074 (0.803 to 1.435)	0.631
≥3	−0.256	0.774 (0.415 to 1.446)	0.422
Mother's education (years)			
≤9		1	
9–12	0.055	1.057 (0.760 to 1.469)	0.741
>12	0.277	1.319 (0.917 to 1.898)	0.136
Father's education (years)			
≤9		1	
9–12	0.305	1.357 (0.963 to 1.912)	0.081
>12	0.407	1.502 (1.052 to 2.144)	**0.025**
Quality of life			
Worse		1	
Poor	−0.493	0.611 (0.313 to 1.192)	0.149
Medium	0.204	1.266 (0.662 to 2.269)	0.517
Good	0.035	1.036 (0.541 to 1.986)	0.915
Better	0.763	2.144 (0.885 to 5.196)	0.091
Annual household income (RMB/y)			
<25 000		1	
25 000–50 000	0.685	1.983 (1.272 to 3.092)	**0.003**
50 001–100 000	0.783	2.188 (1.420 to 3.372)	**<0.001**
100 001–150 000	0.494	1.639 (1.027 to 2.618)	**0.038**
>150 000	0.840	2.317 (1.430 to 3.754)	**0.001**
Obesity	−0.044	0.957 (0.582 to 1.573)	0.862
Children's history			
Have a cold in the last month	−0.179	0.836 (0.637 to 1.098)	0.198
Asthma	−0.719	0.487 (0.289 to 0.822)	**0.007**
Congenital heart disease	−0.161	0.851 (0.111 to 6.502)	0.876
Rhinitis	−0.075	0.928 (0.655 to 1.314)	0.674
Urolithiasis	−0.723	0.485 (0.060 to 3.898)	0.497
Maternal history			
Hypertension	1.125	3.080 (0.423 to 22.413)	0.267
Hepatitis B	−0.318	0.728 (0.260 to 2.038)	0.545
Urolithiasis	1.203	3.331 (0.458 to 24.201)	0.234
Other	−0.425	0.654 (0.297 to 1.437)	0.290
Father's history			
Hypertension	−0.076	0.926 (0.480 to 1.787)	0.820
Diabetes	0.078	1.081 (0.335 to 3.491)	0.896
Hepatitis B	−0.402	0.669 (0.305 to 1.470)	0.317
Urolithiasis	0.981	2.668 (0.841 to 8.459)	0.096
Other	−0.075	0.928 (0.401 to 2.147)	0.861
Preterm birth	−0.141	0.869 (0.527 to 1.432)	0.581
Caesarean section	0.182	1.200 (0.906 to 1.590)	0.204
Gestational hypertension	0.804	2.235 (0.545 to 9.173)	0.264
Gestational diabetes	0.826	2.284 (0.312 to 16.721)	0.416

Significant values in bold.

Table 3 shows the results of the univariate mixed logistic regression analysis for commercial medical insurance. Families with two children were less likely to have CMI for their children (OR 0.726 [95% CI 0.621 to 0.848]) compared with three or more children (OR 0.617 [95% CI 0.421 to 0.904]). In contrast, higher parental education levels were associated with an increased likelihood of CMI enrolment. When either mother or father had >12 y of education, the probability of the child being enrolled in CMI more than doubled (mother: OR 2.324 [95% CI 1.921 to 2.812], father: OR 2.603 [95% CI 2.136 to 3.173]). Similarly, higher household income, especially >150 000 RMB, also increased the likelihood of CMI enrolment (OR 2.441 [95% CI 1.893 to 3.148]). Additionally, childhood obesity was negatively correlated with CMI enrolment (OR 0.708 [95% CI 0.537 to 0.935]). In contrast, positive correlations were observed for children with rhinitis (OR 1.320 [95% CI 1.103 to 1.579]), a history of caesarean delivery (OR 1.427 [95% CI 1.233 to 1.652]) and maternal gestational diabetes (OR 2.363 [95% CI 1.233 to 4.565]).

**Table 3.** Univariate mixed logistic regression analysis of CMI.

Variables	β	OR (95% CI)	p-Value
Age	−0.025	0.975 (0.877 to 1.084)	0.640
Female	−0.096	0.909 (0.796 to 1.038)	0.158
Number of children			
1		1	
2	−0.320	0.726 (0.621 to 0.848)	**<0.001**
≥3	−0.483	0.617 (0.421 to 0.904)	**0.013**
Mother's education (years)			
≤9		1	
9–12	0.539	1.714 (1.428 to 2.056)	**<0.001**
>12	0.843	2.324 (1.921 to 2.812)	**<0.001**
Father's education (years)			
≤9		1	
9–12	0.481	1.617 (1.331 to 1.965)	**<0.001**
>12	0.957	2.603 (2.136 to 3.173)	**<0.001**
Quality of life			
Worse		1	
Poor	−0.085	0.919 (0.625 to 1.351)	0.667
Medium	0.032	1.032 (0.742 to 1.436)	0.851
Good	−0.072	0.931 (0.655 to 1.322)	0.689
Better	0.223	1.249 (0.837 to 1.865)	0.276
Annual household income (RMB/y)			
<25 000		1	
25 000–50 000	0.150	1.161 (0.897 to 1.503)	0.256
50 001–100 000	0.515	1.674 (1.314 to 2.133)	**<0.001**
100 001–150 000	0.585	1.795 (1.367 to 2.356)	**<0.001**
>150 000	0.893	2.441 (1.893 to 3.148)	**<0.001**
Obesity	−0.345	0.708 (0.537 to 0.935)	**0.015**
Children's history			
Have a cold in the last month	0.044	1.045 (0.904 to 1.208)	0.549
Asthma	0.221	1.247 (0.887 to 1.753)	0.204
Congenital heart disease	−0.166	0.847 (0.253 to 2.831)	0.787
Rhinitis	0.278	1.320 (1.103 to 1.579)	**0.002**
Urolithiasis	−1.602	0.202 (0.024 to 1.702)	0.141
Maternal history			
Hypertension	−0.322	0.725 (0.392 to 1.342)	0.306
Diabetes	−0.654	0.520 (0.187 to 1.443)	0.209
Hepatitis B	0.244	1.276 (0.719 to 2.267)	0.405
Urolithiasis	−0.351	0.704 (0.383 to 1.296)	0.260
Other	0.143	1.154 (0.730 to 1.824)	0.541
Father's history			
Hypertension	−0.321	0.726 (0.507 to 1.038)	0.079
Diabetes	0.036	1.037 (0.570 to 1.883)	0.906
Hepatitis B	−0.007	0.993 (0.619 to 1.591)	0.975
Urolithiasis	−0.145	0.865 (0.592 to 1.264)	0.454
Other	−0.150	0.861 (0.538 to 1.378)	0.532
Preterm birth	0.154	1.166 (0.904 to 1.504)	0.236
Caesarean section	0.356	1.427 (1.233 to 1.652)	**<0.001**
Gestational hypertension	−0.188	0.829 (0.498 to 1.379)	0.470
Gestational diabetes	0.860	2.363 (1.223 to 4.565)	**0.010**

Significant values in bold.

### Multivariate mixed logistic model to analyse associated factors of medical insurance

**Figure 2.**
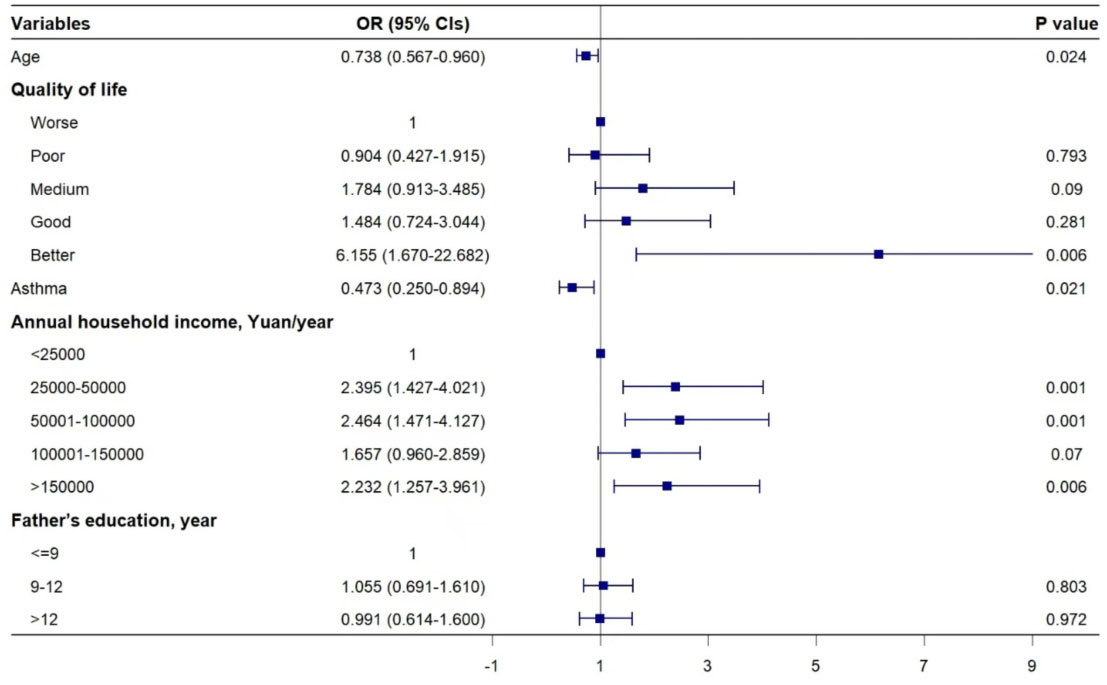
Forest plot for multivariate mixed logistic regression analysis of BMI.

After adjusting for covariates, several factors were found to significantly influence the likelihood of children being enrolled in BMI. Younger age (OR 0.738 [95% CI 0.567 to 0.960]), better quality of life [OR 6.155 [95% CI 1.670 to 22.682]) and high annual household income, especially >150 000 RMB (OR 2.232 [95% CI 1.257 to 3.961]), increased the purchasing of BMI (all p<0.05), and children with a better quality of life were approximately 6 times more likely to be covered by BMI than children with a worse quality of life. Conversely, children with asthma had lower odds of BMI coverage (OR 0.473 [95% CI 0.250 to 0.894]). However, there was no significant association between paternal education level and children's BMI enrolment (Figure 2).

**Figure 3.**
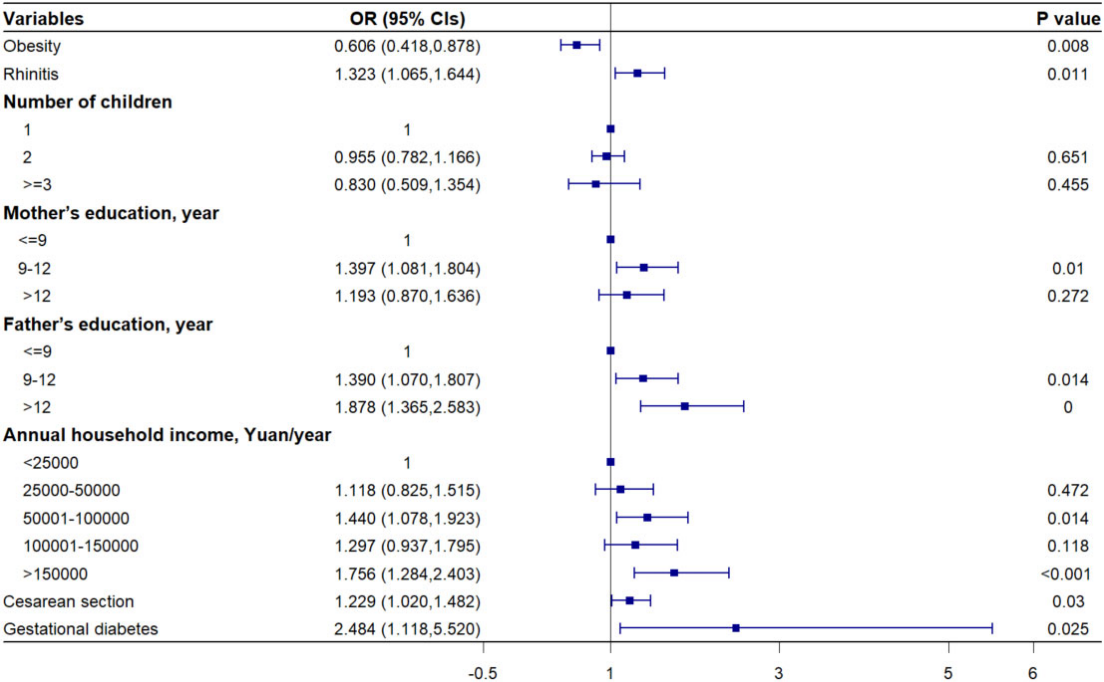
Forest plot for multivariate mixed logistic regression analysis of CMI.

The multivariate mixed logistic model revealed that obesity in children (OR 0.605 [95% CI 0.418 to 0.878]) was a risk factor for being uninsured. In contrast, several factors significantly increased the likelihood of purchasing CMI, including higher parental education levels, children with rhinitis, annual household income >150 000 RMB, a history of caesarean section and maternal gestational diabetes (all p<0.05). Children with a history of caesarean section were 1.229-fold more likely to be insured than those without (OR 1.229 [95% CI 1.020 to 1.482]). Children whose mother had a history of maternal gestational diabetes had approximately twice the odds of being enrolled in CMI compared with their counterparts (OR 2.484 [95% CI 1.118 to 5.520]) (Figure 3).

## Discussion

This study found that children with a better quality of life were more likely to have BMI, while low family income was significantly linked to a lack of either BMI or CMI. Our findings align with existing studies that have identified socio-economic factors as independent contributors to health insurance coverage.^21,22^ Individuals with lower income and fewer resources are economically and socially disadvantaged to a greater extent, which may hinder their ability to afford and enrol in health insurance.^22^

Compared with urban areas, children from rural areas were less likely to obtain insurance coverage, particularly CMI. According to domestic and foreign studies, living in rural areas is significantly associated with lower health insurance coverage.^15,31^ This disparity may be attributed to economic factors, conceptual factors, the cost of medical insurance and the availability of insurance services. One study highlighted that illness was a major contributor of poverty in rural China, with poor health, poverty and low income mutually reinforcing each other.^23^ Therefore, it is significantly necessary to improve rural children's participation in medical insurance. To promote children's health, the government and insurance companies should implement stricter health insurance policy supports targeting rural populations. Our research also found that children with higher-educated parents are more likely to purchase CMI. Individuals with higher levels of education tend to have greater health literacy, better understand the benefits of insurance and often have higher socio-economic status, hence there was a direct correlation between education and medical insurance needs.^32,33^ Furthermore, educated individuals may be more familiar with insurance principles and reimbursement processes.^34^ A longitudinal study of approximately 120 000 children in the USA showed that children's insurance coverage and type are correlated with their parents’ insurance status.^20^ Similarly, DeVoe et al.^19^ found that children whose parents purchase insurance are more likely to be insured themselves. Therefore, raising awareness about health insurance in relatively backward areas is essential, including educating the public on the definition of insurance, coverage, reimbursement processes and the availability of health services.

Our finding on rhinitis was consistent with a South Korean study, which showed that children with chronic illnesses are more likely to purchase private insurance.^16^ Also, a meta-analysis of adults suggested that chronic diseases contribute to increased medical insurance enrollment.^34^ However, we found that asthma was a risk factor for lacking BMI in children, while obesity was a risk factor for lacking CMI. Insufficient disease coverage and low reimbursement expenses for BMI may partially explain why children with asthma are less likely to purchase BMI. Another possible explanation was that inadequate health insurance resulted in low availability of healthcare and poor health outcomes.^35^ Additionally, the low insurance coverage rate among obese children may be partly due to insufficient awareness of the diagnosis and risks associated with obesity. Recent evidence suggests that medical insurance affects unhealthy lifestyles, leading to increased obesity.^36^ Therefore, it is of great significance to strengthen research on medical insurance coverage rates for unhealthy or subhealthy children and the health benefits provided by medical insurance.

In the multivariate model, perinatal factors influencing participation in CMI included a history of caesarean section and maternal gestational diabetes. Although there are no studies currently that explore the relationship between medical insurance and perinatal factors, substantial evidence indicates that gestational diabetes increases the long-term risk of childhood complications such as obesity, impaired glucose metabolism and cardiovascular disease.^26^ Similarly, caesarean section was associated with increased chronic health conditions, including obesity, allergies and asthma.^27^ These adverse perinatal factors may negatively impact children's health outcomes, which could explain why they increase the likelihood of children being insured.

Achieving universal health coverage is China's ambition, but gaps remain, particularly for children. In this study we found that children in Chongqing have not yet achieved full insurance coverage, with a much lower enrolment rate in CMI compared with BMI (29.78% vs 83.29%). In addition, the participation rate of children in BMI was lower than that of the general population (83.29% vs 95%). CMI emerged in China in the 1980s as a crucial supplement to BMI, facilitating individuals in affording medical expenses beyond BMI's scope. Nonetheless, participation of CMI among Chinese residents remains very low, which may be potentially attributable to several factors, including ambiguous market positioning, relatively high premiums and overlapping service scopes with BMI.^37^ A study in China showed that since the COVID-19 epidemic, there has been a significant increase in the demand for CMI, with medical insurance premiums rising by 38.86% year-on-year in the first half of 2020. Perhaps this surge in interest presents an opportunity to improve the status of children's health insurance.^38^ However, as Chen et al.^10^ noted, most regions in China have yet to design and implement insurance programs specifically for children, while developed countries such as the USA have implemented children's health insurance programs.^7,39^ To address this gap, greater emphasis should be placed on expanding coverage and increasing participation rates in children's insurance during the next round of China's healthcare system reform. This could be accomplished by formulating medical insurance policies tailored specifically for children, with strong support from both the government and insurance companies.

### Limitations and strengths

Our research has the following strengths. First, it contributed to the evidence base on children's health insurance in economically disadvantaged regions of southwest China. Second, several chronic diseases were considered in this study due to the low prevalence of severe diseases. Furthermore, we took into account the influence of parental health and perinatal factors on medical insurance enrolment. However, there are also some limitations. First, as a cross-sectional study, it cannot establish a causal relationship between influencing variables and insurance participation. Second, we did not investigate the relationship between medical insurance status and healthcare utilization in children. Third, the study does not comprehensively explore all types of common chronic diseases in children.

### Conclusions

In this study, we found that the participation rate of CMI among children in Chongqing was notably lower than that of BMI, particularly in rural areas. Additionally, children's BMI enrolment lagged behind the national average. Our analysis identified several factors associated with limited access to BMI, including older age, asthma, poor quality of life and low family income. Conversely, factors such as rhinitis, an annual household income >150 000 RMB, higher parental education levels, a history of caesarean delivery and maternal gestational diabetes increased the likelihood of children's enrolment in CMI. In conclusion, the goal of universal health insurance coverage for children in Chongqing has not yet been achieved. To ameliorate the insufficient and imbalanced status of children's insurance, sustained efforts are necessary. This involves exploring the associated factors, developing targeted insurance plans for high-risk paediatric populations and strengthening policy support, especially for economically disadvantaged rural areas in southwest China.

## Data Availability

The datasets of the current study are available from the corresponding author upon reasonable request.
